# Is It Possible to Find Something Positive in Being Confined Due to COVID-19? Implications for Well-Being

**DOI:** 10.3390/ijerph17239087

**Published:** 2020-12-05

**Authors:** M. Dolores Merino, Coral Oliver-Hernández, M. Dolores Vallellano, Inmaculada Mateo

**Affiliations:** 1 Departamento de Psicología Social, del Trabajo y Diferencial, Facultad de Psicología, Campus de Somosaguas, Universidad Complutense de Madrid, 28223 Madrid, Spain; maoliver@ucm.es (C.O.-H.); lolavall@ucm.es (M.D.V.); 2 Escuela Andaluza de Salud Pública, 18080 Granada, Spain; inmaculada.mateo.easp@juntadeandalucia.es; 3 CIBER de Epidemiología y Salud Pública (CIBERESP), 28029 Madrid, Spain

**Keywords:** COVID-19, quarantine, positive affect, negative affect, well-being

## Abstract

In relation to COVID-19, little research has focused on the study of variables that affect well-being during this pandemic. The purpose of this research is twofold: (1) to find out if people experiencing a quarantine are able to appreciate any positive aspects of it, and to analyze how these responses are categorized and (2) to check if there are differences in levels of well-being according to these categories. There were 243 representative participants of the Spanish population according to sex, age, and educational level. The methodology was mixed, qualitative for the first objective and quantitative for the second. The first used the Thematic Network, while the second used logistic regression. From the qualitative analysis, three major categories were extracted: intrinsic orientation, extrinsic orientation, and empty orientation. The quantitative results showed a clear advantage in well-being for the intrinsically oriented group. The group of intrinsic orientation presented a better coping ability while the group of extrinsic orientation was as little adaptive as the group of empty orientation. Recommendations are extracted from the results.

## 1. Introduction

Coronavirus disease 2019 (COVID-19), caused by SARS-COV-2, is the physical health problem having the greatest global impact in recent decades [1]. Isolation measures as a form of effective disease control are not new, and several have been written about throughout history [2]. Today, we are facing one of the worst pandemics of all time, with millions of cases of people infected and dying in more than 188 countries [1]. Given this situation, drastic measures have been implemented around the world aimed at containing mass contagion. Situations of social isolation and quarantine are absolutely exceptional, and most people have never experienced this. Prior studies carried out under similar conditions show its great psychological impact. For example, the study conducted by Reynolds et al. [3] showed that 20% of the population experienced fear, 18% nervousness, 18% sadness, and 10% guilt, and few positive feelings were reported, only 5% reported feelings of happiness and 4% reported feelings of relief. Qualitative studies also identified confusion and anger [4], fear and feeling blocked [5], and anxiety-induced insomnia [6]. Moreover, people who had been subject to quarantine continued to have high levels of stress and worrying levels of psychological distress one year after being exposed to the situation [7].

Different studies [8] show how emerging mental health problems related to this global event can become lasting health problems with serious consequences. We are living in a unique situation, with social isolation and where we are also constantly exposed to data on new deaths or infection rates from the pandemic around the world, which are constantly increasing. This is logically linked to different disorders such as fear, anxiety, and depression [8,9], along with states and conduct that can lead to behaviour that is risky for public health, such as increased alcohol and tobacco use and social isolation [10]. In a study conducted in Spain [11], results similar to those described were reported, but with lower levels of stress, anxiety, and depression. This could be because the study was done at the beginning of the health. 

Although the proliferation of research on the psychological effects of COVID-19 on the population increases every day, little research has focused on studying the variables affecting well-being during this pandemic. In general, studies on well-being can be classified into two different but interrelated perspectives: hedonic well-being and eudaimonic well-being. The first, or subjective well-being, refers to the balance between positive and negative affect (affective component) as well as to the overall assessment of one’s own life (cognitive component). The second, or psychological well-being, identifies happiness with personal realization and puts its focus on the human potentialities that allow us to function positively and “flourish” as people [12,13]. 

In the relationship between COVID-19 and well-being, the small amount of research found has focused on studying the association between fear of getting the illness and well-being, measured in different ways. For example, Lu et al. [14] found that fear of getting the disease was associated with less happiness. Additionally, a positive attitude toward COVID-19 was associated with greater happiness and lower depressive symptoms. In turn, the results from Bakioğlu et al. [15] suggest that intolerance to uncertainty, depression, anxiety, and stress play a mediating role in the relationship between fear of getting COVID-19 and positivity, measured as a combination of optimism, self-esteem, and overall life satisfaction [16]. In research by Satici et al. [8], the relationship between intolerance of uncertainty and mental well-being was studied, and they found this to be mediated by rumination and fear of getting COVID-19. In these investigations, we found consistent results despite the use of different measures of well-being. Sweeny et al. [17] investigated the role of flow. Flow is understood as a state in which the person is completely committed to an activity, during it, loses track of time and experiences enormous satisfaction. In addition, the person uses their skills and abilities to the extreme [18]. Sweeny et al. [17] found that the relationship between the length of the quarantine and well-being was modulated by flow. According to the authors, getting involved in flow-inducing activities can be an effective remedy against the pernicious effects of quarantine. This is because, among others, it prevents boredom and helps time pass faster while, at the same time, doing an intrinsically rewarding activity, thus proving to be an effective coping strategy in the event of a pandemic. 

Our research is related to this studies that attempt to analyze well-being during the quarantine due to COVID-19. In our case, the question was whether people could find something positive in being confined in their homes, due to mandatory quarantine. As far as we know, to date, this question has not been investigated. Mandatory confinement can be a sign of the severity of the infection and is associated with greater discomfort than voluntary confinement [14]. In addition, confinement isolates us but also brings us together, gives us time for the family, for things we have pending, for reflection. It is conceivable that people who are able to find something good in confinement will be predisposed to coping better with it and, consequently, will experience greater well-being during confinement. It is important to understand these issues when developing small interventions that make it more likely that people in quarantine will increase their levels of well-being. In line with the above, the specific objectives of this research are:

1. To understand if people who are experiencing a situation of confinement are capable of appreciating positive aspects in it and, in this sense, to identify which categories emerge from their responses.
2. To analyze whether there are differences in subjective and psychological well-being in the categories identified through objective 1.

## 2. Materials and Methods

The method used was mixed: qualitative to respond to the first objective (Part 1) and quantitative to respond to the second objective (Part 2). 

To respond to the first objective, we used a qualitative method because [19]: (1) It is inductive, going from data to theory, rather than the other way round, non- hypothesis, or any type of “a priori”. In the first objective, we want to investigate if people is able to find something good in being lockdown. Therefore, we do not start from any premise, but try to arrive at it, based on the subjects’ perceptions of their quarantine experience. (2) It is based on generation, and not on verification, which is to say, it is based on the analysis of applied techniques (groups meeting, in-depth interviews, texts analysis, etc.), whereby we seek to discover categories, patterns or constructs that have a certain transferability. In our study, based on the responses given to the open question “Considering your particular case, what is the best thing about this situation?” (the situation refers to confinement because of COVID-19), we want to discover the fundamental dimensions or categories related to their answers. (3) Related to the previous point, qualitative research does not enumerate, but builds categories that emerge from the analysis of the data and that was the last purpose of the first objective. 

To respond the second objective, we used a quantitative transversal method with the purpose to understand which of the categories identified before were better predicting well-being under lockdown due to COVID-19

### 2.1. Participants and Sample

Using a non-probability sampling procedure called snowball or chain sampling [20], the participation of 839 people in the study was achieved. Nevertheless, this sample was overrepresented in terms of sex, age, and education level. To avoid the consequent bias, a subsample of 244 participants was randomly extracted from the previous one considering the Spanish population distribution according to the data of the National Institute of Statistic [21] for those variables (see Table 1). The procedure used were next: the initial participants were coded with 3 digits that represent sex, age, and education, respectively. Two sex categories, three age levels and two levels of studies form 12 groups of individuals. Excel random function were used to select a number of individuals for each group according to the percentage of Spanish population for such groups.

### 2.2. Data Collection

An ethical committee from a Spanish public university (Ref. 2019/20-030) approved the research. An online questionnaire, which contained both the qualitative and quantitative components for data collection, was designed through the Google Surveys application. The link to the questionnaire was launched via social media, and subjects interested in participating accessed the questionnaire after reading and accepting the informed consent form. Responses were obtained in the last week of March.

### 2.3. Measures

Qualitative component

To explore the insights for the qualitative objective, the following open question was asked:

“Considering your particular case, what is the best thing about this situation?” (the situation refers to confinement because of COVID-19).

Quantitative component

There were three measures used for the quantitative component of the questionnaire:

#### 2.3.1. The First: Overall Measure of Perceived Happiness

A question used by the Centre for Sociological Research [22] was adapted to measure the levels of happiness perceived by the population. Specifically, the question was: “Thinking about yourself, at this moment how happy are you feeling?” There were five alternative responses on a Likert-type scale. This question has been used as a measure of subjective well-being, cognitive component. 

#### 2.3.2. The Second: Positive and Negative Affect Schedule (PANAS Scale)

We used the updated Spanish adaptation [23] of the original scale from Watson et al. [24]. This scale has 20 items with five Likert-type response points and two dimensions: positive affect and negative affect. This scale has been widely used and adapted to many countries [23] and it has been used as a measure of subjective well-being (affective component).

#### 2.3.3. The Third: Positive Psychological Functioning Scale (PPF)

This scale is a measure of psychological well-being with five Likert-type response points. It consists of 33 items that group together 11 psychological resources, which in turn form a second-order factor, which is what provides the scale’s name [25]. It has reliable psychometric properties and has been adapted to other countries [26,27]. In the present study, the scale exhibited very high internal consistency with a Cronbach’s alpha of 0.94.

### 2.4. Analysis

#### 2.4.1. Analysis Strategy: Objective 1

To analyze answers to the question, “Considering your particular case, what is the best thing about this situation?” (confinement because of COVID-19), the Thematic Network proposed by Attride-Stirling [28] was used as a tool to analyze texts consisting of three phases: (1) Identification of basic themes (Basic Theme), (2) Organization of basic themes (Organizing Theme), and (3) Identification of global theme (Global Theme). The technique was adapted to the characteristics of this study as follows: (1) To identify the basic themes: (1.1) Reading and rereading the 244 responses given by the participants. (1.2) Identification in each response of the key topic; that is, as a matter of priority, contributed to attributing meaning to each of the 244 answers given. For example, the response from Subject 30 was to be with the entire family. The key theme in this case is family. (2) For the organization of themes: Comparison of similarities and differences between the meanings of the different basic themes found and grouping according to their similarities. (3) Identification of the global theme in the groupings from step 2 that gives rise to the category. 

It should be noted that in order to avoid subjectivities of the authors and to triangulate the result, all steps were discussed in analytical meetings by the four researchers until reaching inter-judge agreements. 

#### 2.4.2. Analysis Strategy: Objective 2

Descriptive statistics were calculated for each of the well-being dimensions, utilized according to the type of orientation found in the qualitative analysis (intrinsic, extrinsic, and empty or nothing). A logistic regression analysis was later done comparing people with intrinsic orientation with those who have extrinsic orientation and with those having empty orientation, and the group having extrinsic orientation versus empty orientation in psychological and subjective dimensions of well-being.

## 3. Results

### 3.1. Results of Objective 1

From analysis of the responses given by the 244 participants, seven categories surfaced that we have arranged here from highest to lowest of occurrence and which structured 100% of the responses. It should be said that only 5.7% of total responses had double references. For example: “Spend more time with family and telecommuting.” In these cases, only the first reference was classified in order to not have repeated dimensions. 

The first category was called “creating closer ties with the family”. This dimension brought together 24.69% of the answers given. By analyzing this information, it is inferred that the situation of confinement in homes has made it possible for family members and people who cohabitate to spend more time together, creating closer ties, strengthening their union and the enjoyment of living together, something they could not do in a normal situation. Some examples that illustrate this category would be: “Enjoying the family more” (Subject 100). “I’m spending more time with my parents and playing a lot with them” (Subject 132). “Family togetherness” (Subject 161). “Being with your loved ones” (Subject 167). “Having more time to be with my son” (Subject 192). 

The second category was called “personal development” and brings together a set of responses regarding with personal growth. There were 22.2% in this group. Thus, included in this category were responses mentioning reflection and introspection, returning to essential values, and learning. Some examples of reflection and introspection of are: “You value things that didn’t matter before” (Subject 9). “Inner reflection” (Subject 42). “Time to think and reconsider what is urgent and what is really important” (Subject 92). “Self-analysis” (Subject 158). In regard to values: “Resilience” (Subject 29). “Civic response” (Subject 41). “Togetherness” (Subject 59). “Seeing there is increasing awareness, more solidarity” (Subject 141). With respect to learning: “Time to learn new things” (Subject 13). “Learning to do things better” (Subject 220).

The third category was called “time” (17.3% of the responses). This dimension gathered answers related to the possibility of taking advantage of the time the confinement gave them to devote themselves to doing things they had pending, to devote more time to themselves, and to enjoy time for itself. Some examples of this category included: “Having time to do things I don’t usually do” (Subject 221). “Having more time for myself without outside pressures” (Subject 179). “Having free time” (Subject 76).

The fourth category was called “nothing”. 13.9% of the subjects found nothing positive in the confinement. Some examples are “Nothing” (Subject 2). “I don’t see anything positive” (Subject 113).

The fifth category was called “health”, and it groups together a whole series of responses that refer to taking care of your health by isolating, which makes it possible for subjects to sleep more, not have the usual stress, and to feel protected from infection. Some 10.28% of the answers belonged to this category. Some examples here are: “Relax” (Subject 114). “Don’t have to wake up early” (Subject 125). “Having a lower risk of infection” (Subject 222).

The sixth category, called “telecommuting and study”, was very small (7.4%) and grouped together references to the perceived advantage in the change of daily obligations, whether work or study, due to not having to be physically present. Some examples were: “Telecommuting” (Subject 16). “Not having to be physically present at work” (subject 28). “Cyber-classes” (Subject 134). “More time to focus on studying” (Subject 128).

The seventh category, the last, was called “miscellaneous”. A few responses (4.5%) were grouped here that did not fall into any of the six categories mentioned above. They mentioned issues such as the following: “Saving” (Subject 4). “My location” (Subject 63). “Going out to walk the dogs” (Subject 228). 

Analysis of these categories reveals three global themes: Intrinsic orientation, extrinsic orientation, and empty orientation. In intrinsic orientation we group those categories that, due to their content, refer to positive aspects that are valued for themselves. Categories forming part of this large group would be strengthening family ties, personal development, and time. In extrinsic orientation we include those categories that, from their analysis, refer to practical issues directly associated with the state of confinement itself, such as preserving health, telecommuting, or miscellaneous issues such as savings or walking the dog. Finally, empty orientation would constitute the smallest segment, made up of the category called nothing, in which responses from subjects who did not perceive any positive aspect in the confinement were grouped together. 

### 3.2. Results of Objective 2

Table 2 shows the median and standard deviation of each of the dimensions on well-being analysed in total and according to the type of orientation (intrinsic, extrinsic, and empty). 

It has also been shown that when intrinsic orientation model is compared with an extrinsic orientation, and intrinsic or extrinsic orientation is compared with empty orientation, they differ in their levels of subjective and psychological well-being. The results of this analysis can be found in Table 3. Results from PPF Scale [25] found people with intrinsic orientation have higher levels of psychological well-being than those with extrinsic orientation (OR = 1.9; CI 95% = 1.16–3.15). In addition, those with an intrinsic orientation have higher levels of overall happiness (OR = 2.05; CI 95% = 1.22–3.43) and psychological well-being (OR = 2.41; CI 95% = 1.35–4.32) than those who do not perceive any opportunity in a situation of confinement (empty orientation). 

The type of orientation is not associated with either positive or negative affect. Although it is shown that the intrinsic orientation group scores higher in positive affect, close to significance when compared with the empty orientation group (*p* = 0.062), this result should be considered tendentially. 

Nor are there differences in levels of subjective or psychological well-being between those with an extrinsic orientation and those having empty or not orientation. 

## 4. Discussion

Although research systematically shows that confinement is associated with discomfort, and it is greater the longer the confinement lasts [2], most people are able to find something positive in being confined. More than half show an intrinsic orientation strategy (64.2%); meaning, taking advantage of confinement to (1) enjoy being with the family; (2) personal development, appreciation of social values, or learning, and (3) enjoy available time, whether for yourself, to do things you have pending, or to enjoy free time at home. 

Almost a quarter (22.2%) follow an extrinsic oriented strategy. This means they are able to appreciate something positive with confinement. However, it is closely linked to the advantages of confinement itself, such as (1) taking care of health through isolation, which allows you to be more protected from being infected and to rest more; (2) telecommuting and tele-studying, as in many cases being physically present was prohibited; and (3) diverse issues such as the savings associated with lower consumption due to confinement. 

Only 13.9% belonged to the empty orientation group. This group included people who were unable to find something positive in being confined. 

When we compare the three groups with respect to the subjective and psychological levels of well-being experienced, it is interesting to see that the group with higher levels of well-being is the one with intrinsic orientation. This group experiences higher levels of psychological well-being, both when compared with the extrinsic orientation group and with the empty orientation group. This is a very consistent result, as these people have been able to identify positive aspects in confinement that are related to factors clearly linked to psychological well-being such as: positive relationships with others [29,30], personal growth, and taking advantage of time [18]. Nevertheless, what the extrinsic orientation group identifies are factors that do not depend on action or personal will but rather on the context experienced. However, there are no differences in subjective well-being when comparing the intrinsic and extrinsic orientation groups. 

Furthermore, when comparing the intrinsic orientation group with the empty orientation group, we found that the first one to show higher levels of psychological well-being and subjective well-being. It should be noted, however, that subjective well-being shows higher levels of positive affect (at the limit of significance) and in perception of overall happiness, but not of negative affect. In fact, there are no differences in negative affect in any of the groups. Thus, although people can find advantages in lockdown that allow them to develop more adaptive coping, they experience greater well-being, as is the case with the intrinsic orientation group. This does not mean they are disconnected from reality and do not suffer because of it. In other words, they have been able to develop an adaptive coping strategy. Which cannot revolve around the problem, as its modification is beyond the person’s control and does not imply a positive reinterpretation of it [31]. However, it does imply an appreciation of the positive such an anomalous and extraordinary situation can offer and to take advantage of it. This response implies a clear advantage for the intrinsic orientation group, as our results demonstrate. However, the strategy followed by the extrinsic orientation group is just as non-adaptive as that of the empty orientation group, as they do not show a difference in their levels of subjective and psychological well-being. 

Finally, the differences in well-being found at the quantitative level in the three groups triangulate the qualitative analysis, thus enhancing the validity of the findings [32]. If the categories had been erroneously configured, we would have been found no differences. Moreover, the results found in the quantitative analyses triangulated [32] the classification obtained in the qualitative analysis, giving coherence to the proposed objectives.

### 4.1. Practical Applications

Given our results show that intrinsically oriented people are better at coping with the quarantine, we are of the opinion that guiding others the strategies they have followed may help them in similar situations in the future. In this regard, the recommendations inferred from the results would be:

1. Cultivate conscious appreciation of the social and individual values that arise in such dramatic situations, such as solidarity, connection, civility, or resilience.
2. Devote time to personal reflection and introspection as an opportunity to improve as individuals.
3. Learn to appreciate the truly important things in life, which in the daily hustle and bustle go unnoticed.
4. Take advantage of the moment to learn about new things that really motivate us but due to lack of time we had never pursued before.
5. Take advantage of and appreciate the time available to do things we have been meaning to do related to learning, reading, taking care of the home or self-care and, in general, resolving pending issues.
6. In the case of structured family that lives together during the quarantine, taking advantage and enjoying the time they have together that daily life during normal situations takes from them, and doing activities in common that distract them, letting you have fun and strengthen ties.

### 4.2. Future Research Lines

It would be interesting to test the strategies identified by practicing them in a possible future situation of confinement with people from the empty and extrinsic groups, and to observe whether their levels of well-being increase. Of course, adapting each strategy to the characteristics of each person, making pre- and post-test measures, and comparing with a control group belonging to the same groups and having the same sociodemographic characteristics, and comparing with the intrinsic group. These results would allow us to clarify whether such strategies are truly effective in situations of quarantine.

### 4.3. Limitations

This is a cross-sectional study with a statistically sufficient sample size, but not representative of the Spanish population. However, the population distribution is respected in terms of sex, age, and educational level (in two levels). It would have been interesting to have longitudinal data that allowed us to understand how the variables studied evolve as the confinement lengthens.

## 5. Conclusions

According to the results, we can conclude that those with intrinsic orientation are shown to be the most adaptive in the face of experiencing well-being during the quarantine. Nevertheless, the people with extrinsic orientation is just as non-adaptive concerning well-being as those with empty orientation. At the same time, the fact that some groups show lower levels of subjective or psychological well-being does not imply that they are unable to experience positive affect. Finally, it is important to remark that being able to appreciate positive aspects in the confinement does not imply being oblivious to the dramatic situation experienced and not suffering from it.

## Figures and Tables

**Table 1:** Sample distribution by sex, age, and educational level.

Sex	Age	University Education	Percentage of Participants in the Study	*n*
Men	<35	No	7%	17
		Yes	4%	11
	35–54	No	12%	24
		Yes	7%	18
	>55	No	13%	33
		Yes	5%	12
Total men			48%	115
Women	<35	No	6%	14
		Yes	6%	14
	35–54	No	11%	27
		Yes	8%	20
	>55	No	16%	41
		Yes	5%	13
Total women			52%	129
Total			100%	244

Note: The population data in each stratum are taken from the INE (2019).

**Table 2:** Descriptive statistics and reliability of measures of psychological well-being (PPF) and subjective well-being (PANAS and overall happiness) according to the type of orientation.

Measures					Intrinsic Orientation			Extrinsic Orientation			Empty Orientation		
	*n*	*M*	*SD*	α	*n*	*M*	*SD*	*n*	*M*	*SD*	*n*	*M*	*SD*
PPF	244	3.75	0.65	0.942	156	3.85	0.56	54	3.60	0.75	33	3.48	0.80
PANAS+	244	3.30	0.75	0.906	156	3.38	0.72	54	3.18	0.77	33	3.15	0.85
PANAS−	244	2.39	0.76	0.806	156	2.40	0.78	54	2.42	0.76	33	2.30	0.73
Overall happiness	244	3.00	0.76		156	3.10	0.73	54	2.89	0.84	33	2.70	0.73

**Table 3:** Regression analysis of the association between type of orientation and measures of well-being.

Well-being Measurement	OR *	CI 95% **	*p*
Intrinsic Orientation versus Extrinsic Orientation			
PPF	1.91	1.16–3.15	0.011
PANAS+	1.45	0.95–2.22	0.081
PANAS−	0.96	0.64–1.44	0.867
Overall happiness	1.42	0.94–2.15	0.088
Intrinsic Orientation versus Empty Orientation			
PPF	2.41	1.35–4.32	0.003
PANAS+	1.59	0.97–2.60	0.062
PANAS−	1.18	0.71–1.34	0.511
Overall happiness	2.05	1.22–3.43	0.006
Extrinsic Orientation versus Empty Orientation			
PPF	1.22	0.69–2.15	0.492
PANAS+	1.11	0.64–1.91	0.706
PANAS−	1.24	0.68–2.23	0.475
Overall happiness	1.36	0.77–2.37	0.273

* OR = Odds Ratio; ** CI = Confidence Interval.
